# Network-Based Discovery of Opioid Use Vulnerability in Rats Using the Bayesian Stochastic Block Model

**DOI:** 10.3389/fpsyt.2021.745468

**Published:** 2021-12-17

**Authors:** Carter Allen, Brittany N. Kuhn, Nazzareno Cannella, Ayteria D. Crow, Analyse T. Roberts, Veronica Lunerti, Massimo Ubaldi, Gary Hardiman, Leah C. Solberg Woods, Roberto Ciccocioppo, Peter W. Kalivas, Dongjun Chung

**Affiliations:** ^1^Department of Biomedical Informatics, The Ohio State University, Columbus, OH, United States; ^2^Department of Neuroscience, Medical University of South Carolina, Charleston, SC, United States; ^3^School of Pharmacy, University of Camerino, Camerino, Italy; ^4^School of Biological Sciences, Queen's University Belfast, Belfast, United Kingdom; ^5^Department of Internal Medicine, Wake Forest University School of Medicine, Winston-Salem, NC, United States

**Keywords:** clustering, community detection, Bayesian model, opioid use disorder, network analysis, stochastic block model

## Abstract

Opioid use disorder is a psychological condition that affects over 200,000 people per year in the U.S., causing the Centers for Disease Control and Prevention to label the crisis as a rapidly spreading public health epidemic. The behavioral relationship between opioid exposure and development of opioid use disorder (OUD) varies greatly between individuals, implying existence of sup-populations with varying degrees of opioid vulnerability. However, effective pre-clinical identification of these sub-populations remains challenging due to the complex multivariate measurements employed in animal models of OUD. In this study, we propose a novel non-linear network-based data analysis workflow that employs seven behavioral traits to identify opioid use sub-populations and assesses contributions of behavioral variables to opioid vulnerability and resiliency. Through this analysis workflow we determined how behavioral variables across heroin taking, refraining and seeking interact with one another to identify potentially heroin resilient and vulnerable behavioral sub-populations. Data were collected from over 400 heterogeneous stock rats in two geographically distinct locations. Rats underwent heroin self-administration training, followed by a progressive ratio and heroin-primed reinstatement test. Next, rats underwent extinction training and a cue-induced reinstatement test. To enter the analysis workflow, we integrated data from different cohorts of rats and removed possible batch effects. We then constructed a rat-rat similarity network based on their behavioral patterns and implemented community detection on this similarity network using a Bayesian degree-corrected stochastic block model to uncover sub-populations of rats with differing levels of opioid vulnerability. We identified three statistically distinct clusters corresponding to distinct behavioral sub-populations, vulnerable, resilient and intermediate for heroin use, refraining and seeking. We implement this analysis workflow as an open source R package, named mlsbm.

## Introduction

Opioid addiction is a chronic neuropsychiatric disorder characterized by compulsive drug taking and relapse, despite efforts to remain abstinent. Opioid use disorder (OUD) has risen substantially in the United States over the past two decades, for both prescription drugs (1), as well as illicit opioids, notably heroin (2). The parallel rise in both prescription and illicit opioid use and abuse are related to one another, as a majority of heroin users report using prescription opioids prior to heroin use (2–4). Death due to an overdose is also positively correlated between these two opioid classes (2), posing an additional obstacle in addressing the current opioid epidemic. Furthermore, heroin use since 2000 has increased in all demographics, regardless of age, sex or socio-economic status (2, 4), suggesting factors independent of these are contributing to the escalation in OUD. This ubiquitous increase in heroin use and dependence across disparate populations highlights the need to assess how individual variation in multiple behavioral traits may be interacting to contribute to an OUD resilient vs. vulnerable phenotype.

OUD remains such a critical social and personal problem in part because we are limited by current animal models that predict neurological pathologies for OUD. Though animal models capturing individual variation in addiction-related behaviors have greatly contributed to our understanding of drug addiction, most focus on one or two behavioral phenotypes, then apply the power of animal experimentation to uncover circuitry and cellular mechanisms for individual phenotypes. While this approach has greatly enhanced our understanding of how brain circuits and cell signaling mechanisms contribute to specific behavioral phenotypes, OUD is a disorder containing many behavioral traits that may contribute differentially to resilience and vulnerability to drug addiction depending on individual genetics and sociology (5–7). Indeed, the DSM-V diagnostic criteria for OUD is neither meeting a single behavioral criterion nor meeting all criteria, but rather a person needs to meet a subcluster of criteria to be considered diagnostic (5). This diagnostic protocol is employed because of individual differences resulting from the presence of one diagnostically positive trait does not necessarily predicting the presence of another trait. In an effort to more accurately portray the multi-trait nature of substance use disorders (SUDs), some studies have created composite scores consisting of a few traits that are generally summed in a linear manner to create an addiction score (8, 9). Here we propose a different approach to analyzing multiple traits and explore a multidimensional data clustering strategy of seven behavioral traits potentially characteristic of heroin use and seeking in 451 outbred rats, examined in two distinct laboratories, one at the Medical University of South Carolina (MUSC) in the USA and the other at the University of Camerino (UCAM) in Italy. This approach allows for non-linear relationships between multiple traits to be simultaneously quantified, resulting in clusters of animals that may correspond to overall resilient and vulnerable subgroups.

Various clustering algorithms are available, including k-means clustering (10), hierarchical clustering (11), and finite mixture models (12), among others. However, behavioral studies generate complex multivariate measurements which can make clustering difficult using standard algorithms. Recently, network-based clustering approaches have become popular across multiple disciplines due to their flexibility and applicability to high-dimensional data. For example, in high dimensional single cell genomics studies, these algorithms are employed in multiple software packages for identifying latent cell types such as T and B cells (13). In general, these network-based clustering approaches first construct a similarity network based on observations and then implement a community detection algorithm on this similarity network to identify underlying clusters. As a result, these approaches are less affected by violations of underlying assumptions, such as Gaussianity.

In this paper, we adopt the stochastic block model (SBM), which has strong and rigorous theoretical foundation in statistics literature (14, 15). In essence, the SBM allows for identification of latent communities using a probabilistic model that describes interconnectivity between nodes within and between clusters. In this sense, the SBM may be used as a descriptive tool to assess the presence of distinct latent populations in a data set. The biological utility of such populations may then be determined by investigating the distributions of relevant variables (e.g., heroin consumption) across clusters. While we do not seek to propose a predictive model for opioid vulnerability, the sub-populations identified from our approach may be correlated with data from future studies (e.g., genetic studies) to assess the predictive ability of characteristics that define the identified sub-populations.

Due to its probabilistic nature, the SBM has multiple strengths over deterministic approaches. First, it provides a natural framework for deriving uncertainty measures for identified clusters, which are critical to understanding latent community structure, e.g., understanding gradual changes across multiple latent clusters. Second, using goodness-of-fit measures, the SBM helps selection of the number of clusters, which is a long-standing problem in clustering methodology and not straightforward to address in deterministic algorithmic approaches. Finally, the SBM fits naturally into the Bayesian framework, allowing for incorporation of prior expert knowledge to guide the clustering and the ability to make posterior probability statements about all model parameters (15).

## Materials and Methods

### Experimental Methods

All experimental procedures were approved by the Institutional Animal Care and Use Committee at MUSC and by the Italian Ministry of Health (approval 1D580.18). Procedures abided by the National Institute of Health Guide for the Care and Use of Laboratory Animals and the Assessment and Accreditation of Laboratory Animals Care, as well as the European Community Council Directive for Care and Use of Laboratory Animals.

A total of 600 heterogeneous stock (HS: originally n/NIH-HS) rats bred at Wake Forest University (currently NMcwiWFsm:HS; Rat Genome Database number 13673907) were obtained for these studies. Of these rats, 149 were excluded from final analyses due to death following surgery (*n* = 21), death over the course of training (*n* = 77) or undergoing saline, not heroin, self-administration training (*n* = 51). Final analyses were performed on 451 rats (males, *n* = 238; females, *n* = 213). HS rats were outbred from eight inbred strains and maintained in a way to minimize inbreeding (16), allowing genetic fine-mapping to relatively small intervals (17). Animals were shipped in batches of 40 (20 males and 20 females per site) to either MUSC (USA) or UCAM (Italy) at approximately 5 weeks of age. Upon arrival, animals were pair-housed and left undisturbed in a climate-controlled colony room with a standard 12-h light:dark cycle for 3 weeks prior to the start of testing. Throughout training, rats had *ad libitum* access to food and water. Testing occurred during the dark cycle, between 18:00 and 6:00 h. Heroin hydrochloride supplied by the National Institute on Drug Abuse (Bethesda, MD) dissolved in 0.9% sterile saline was used in these studies.

Following the 3-week acclimation period, rats underwent surgery under isoflurane anesthesia for the implantation of an indwelling jugular catheter. An analgesic (Ketorolac, 2 mg/kg, sc; or Meloxicam, 0.5 mg/rat, sc), and antibiotic (Cefazolin, 0.2 mg/kg, sc; or enrofloxacin, 1 mg/kg, iv), were administered pre-operatively. Rats were given a minimum of 3 days of recovery prior to heroin self-administration training commencing. All testing occurred in standard behavioral testing chambers (MED Associates, St. Albans, VT, USA). Presses on an active lever resulted in presentation of a light and tone cue for 5-s and an infusion of heroin (20 μg/kg/100 μg infusion over 3 s) on a fixed-ratio 1 schedule of reinforcement. At the start of the infusion, the house light also turned off for 20-s signaling a time-out period during which additional presses on the active lever were recorded but without consequence. Presses on the inactive lever were recorded but without consequence. Sessions lasted for 12 h or until 300 infusions were earned. Self-administration occurred Monday-Friday, with one session off per week, for a total of four sessions/week. Following 12 self-administration sessions rats underwent a progressive ratio test whereby the number of presses *p*(*t*) required to receive an infusion increased exponentially after each infusion *t* = 1, …, *T* according to the function *p*(*t*) = 5*e*^0.2*t*^−5 (18). Rats then had three more days of self-administration training to re-establish baseline heroin-taking behavior prior to tests for reinstatement.

At the conclusion of heroin self-administration training, rats underwent a within-session extinction-prime test that lasted for 6 h. The first 4 h were extinction training conditions during which presses on both the active and inactive lever were recorded but without consequence (i.e., active lever presses no longer result in presentation of the light/ tone cues or heroin infusion). With 2 h left in the session, rats were administered an injection of heroin (0.25 mg/mg, sc), and continued testing under extinction conditions. Daily extinction training sessions (2 h) then commenced for 6 consecutive days prior to a test for cue-induced reinstatement. During this test, presses on the active lever resulted in presentation of the light/tone cue and turning off of the house light, but no heroin infusions.

At the conclusion of training, several behavioral measures were selected for clustering analyses to reflect three behaviorally distinct phases of drug addiction: drug-taking (drug reinforced behavior), refraining (drug non-reinforced behavior), and seeking behaviors (both drug reinforced and non-reinforced). Heroin-taking behaviors include total heroin consumption (total μg/kg heroin consumed across the first 12 self-administration training session), escalation of intake (total heroin consumed the first 3 days of self-administration subtracted from the last 3 days; see Supplementary Figure 2 for heroin self-administration acquisition curve), and break point achieved during the progressive ratio test. The break point is the total number of active lever presses the rat is willing to perform in order to receive an infusion of heroin. Refraining behavior consisted of active lever presses during the first 2 h of the within-session extinction-prime test (extinction burst) and the last day of extinction training prior to the test for cue-induced reinstatement (extinction day 6). Two extinction training time points were used as to capture refraining behavior immediately after heroin taking, and following several sessions of non-reinforced seeking prior to cue-induced reinstatement. Heroin-seeking behavior is represented by active lever presses during the heroin-prime and cue-induced reinstatement tests. Active lever presses were used for all variables to maintain continuity in measured behavioral output for each behavior.

### Data Pre-processing

#### Batch Correction for Multi-Site Samples

To analyze the MUSC and UCAM cohorts simultaneously, we first performed a visual inspection of possible batch effects between the two study sites. Specifically, we began by concatenating the raw data matrices from each site into an integrated data matrix, where rows corresponded to individual rats and columns correspond to behavioral measures, as described in section Experimental Methods. Then, to facilitate visualization, we applied the Uniform Manifold Approximation and Projection (UMAP) (19) algorithm to compute 2-dimensional embeddings for each rat. To correct for the apparent batch effect between study sites, we *z*-score transformed each behavioral measure *within study site*. This allowed for analysis of each behavioral measurement on a standardized scale, and, in effect, regressed out unwanted site-specific effects. Distributions of raw behavioral measures (i.e., before z-scoring) are shown in Supplementary Figures 5, 6.

#### Similarity Network Construction

After integrating the behavioral data from each study site as described in section Batch Correction for Multi-Site Samples, we constructed a rat-rat similarity network as follows. First we defined a single parsimonious subset of relevant behavioral measures from the experiments discussed in section Experimental Methods using expert knowledge. Here, the goal was to choose variables that reflected the behavioral propensity of each rat for opioid dependence. Next, we computed the Euclidean distance between each pair of rats using this single parsimonious variable subset. We then formed a rat-rat similarity network, i.e., a collection of nodes and edges, where nodes in the network represent individual rats and edges represent similarities between rats. We placed an edge from each node to its *R* closest other nodes based on the rat-rat distance measures. Here, the number of neighbors *R* is a tuning parameter that controls the density of edges in the similarity network. By default, we adopt the widely used heuristic $R=\sqrt{N}$ (20).

### Stochastic Block Model

To detect communities within the overall rat-rat similarity matrix that might correspond to behaviorally distinct sub-populations, we adopted the Bayesian stochastic block model (SBM), a generative model for network data (15). Let **A** be an *n* × *n* adjacency matrix encoding the rat-rat similarity network among *n* total rats, with *A*_*ij*_ = 1 if rat *i* shares an edge with rat *j* (*i* ≠ *j*), and *A*_*ij*_ = 0 otherwise. For a fixed and pre-specified number of communities, *K*, the SBM assumes


(1) $$A_{ij}|z,\Theta \overset{ind}{\sim }\text{Bernoulli}(\theta_{z_{i},\;z_{j}})\text{ for }i<j=1,\ldots ,n,$$

where *z*_*i*_ ∈ {1, …, *K*} is a categorical indicator variable that denotes the community membership of rat *i*, **z** = (*z*_1_, …, *z*_*n*_), and **Θ** is a *K* × *K* connectivity matrix with elements θ_*rs*_ described in detail below. Equation (1) implies that the probability of an edge occurring between two nodes depends only on the community membership of each node. Thus, all rats belonging to the same sub-population are regarded as *stochastically equivalent*.

While our primary object of inference is the vector of latent community indicators **z**, an advantage of the SBM over other community detection algorithms is its ability to conduct statistical inference on the edge probability parameters θ_*rs*_, for *r* ≤ *s* = 1, …, *K*. By encoding these parameters in a symmetric connectivity matrix **Θ**, we obtain a useful summary of community structure. Here, diagonal elements of **Θ** are within-community edge probabilities, and off-diagonal elements of **Θ** are between-community edge probabilities. In most cases, we expect to find an *assortative* community structure, in which within-community connections are more likely than between-community connections, though the model is capable of detecting *dissortative* community structures as well (21). Thus, in addition to the community labels, the SBM allows us to characterize the global relationships between communities.

Commonly, the SBM as formulated in model (1) is refined to accommodate heterogeneous degree distributions, i.e., *degree correction* (22). Since model (1) assumes that the probability of an edge being place between two nodes only depends on the community membership of the nodes, it is not suitable for networks in which each node may have varying degree, that is, the number of edges connected to it. However, as described in section Similarity Network Construction, our workflow relies on construction of a nearest neighbors network, in which each node, by definition, will have exactly *R* edges, thus degree correction is not necessary.

We estimate parameters of the SBM using a fully Bayesian approach by assigning prior distributions to all unknown model parameters. We select conjugate priors to obtain closed-form full conditional distributions of all model parameters, which in turn allows for straightforward Gibbs sampling. First, for the cluster indicators *z*_1_, …, *z*_*n*_, we assume a conjugate multinomial-Dirichlet prior with $z_{i}\overset{iid}{\sim }\text{Categorical}\left(\pi \right)$ for *i* = 1, …, *n*, and **π** ~ Dirichlet(α_1_, …, α_*K*_), where **π** = (π_1_, …, π_*K*_) control the number of nodes in each community, i.e., the community size. Similarly, we adopt a conjugate beta-Bernoulli prior for **Θ** by letting $\theta_{rs}\overset{iid}{\sim }\text{Beta}\left(\beta_{1},\beta_{2}\right)$ for *r* < *s* = 1, … *K*. By default, we opt for weakly informative priors by setting α_1_ = α_2_ = … = α_*K*_ = 1 and β_1_ = β_2_ = 1 (23).

### Posterior Inference

We implement parameter estimation using Gibbs sampling, as detailed in the Supplementary Material. A critical step of our proposed workflow for identifying behavioral sub-populations in rats is the choice of *K*, i.e., the number of communities. Since the choice of *K* should consider both expert knowledge and evidence from the data, we refrain from proposing a “one size fits all” globally optimal method for choosing of *K*. Instead, in section Results we discuss how Bayesian Information Criterion (BIC) (24) can be used in conjunction with biological knowledge to make informed choices for *K*.

Label switching is an issue encountered in Markov chain Monte Carlo (MCMC) methods, such as the Gibbs sampler proposed above, wherein the model likelihood is invariant to permutations of a latent categorical variable such as **z**. As a result, we may observe natural permutations of **z** over the course of the MCMC sampling that cause the estimates of all other community-specific parameters to be conflated, thereby jeopardizing the accuracy of model parameter estimates. This problem is exacerbated when communities are not well-separated. Previous works have attempted to address the issue by re-shuffling posterior samples after the sampling has completed (25). However, these post-sampling methods rely on prediction and thereby are fallible to prediction error. To address label switching, we adopt the canonical projection of **z** proposed by (26) in the context of Bayesian SBMs, in which we restrict samples of **z** to the canonical sub-space Z={z:ord(z)=(1,...,K)}. In other words, we permute **z** at each MCMC iteration such that community 1 appears first in **z**, community 2 appears second in **z**, *et cetera*. Finally, we choose as our final estimate of **z** the maximum *a posteriori* (MAP) estimate of **z** across all post-burn MCMC samples (23).

### Continuous Phenotyping

While the SBM presented thus far assumes that the overall experimental cohort can be decomposed into a fixed number of discrete communities, where each experimental unit (e.g., rat) is assigned to exactly one community, often interest lies in further differentiating members within a community in a more continuous fashion. Indeed, a core benefit of the Bayesian SBM is that the discrete model structure may be augmented using uncertainty measures, i.e., a quantification of our inferred level of confidence in each estimated model parameter. For instance, let $\hat{\text{z}}=\left({ẑ}_{1},...,{ẑ}_{n}\right)$ be the posterior estimate of the true community labeling vector **z** obtained from the MCMC estimation procedure described in the Supplementary Material. Letting *s* = 1, …, *S* index the post burn-in MCMC iterations, we may quantify the uncertainty in each estimate *ẑ*_*i*_ as


(2) $$\begin{array}{l} u\left({ẑ}_{i}\right)=1-\frac{1}{S}\overset{S}{\underset{s\;=\;1}{\sum }}I\left({ẑ}_{i}^{\left(s\right)}={ẑ}_{i}\right)\;\text{for }i=1,...,n, \end{array}$$

where ${ẑ}_{i}^{\left(s\right)}$ is the estimate of *z*_*i*_ at MCMC iteration *s*, and $I\left({ẑ}_{i}^{\left(s\right)}={ẑ}_{i}\right)$ is the indicator function equal to 1 if ${ẑ}_{i}^{\left(s\right)}={ẑ}_{i}$ and 0 otherwise. In words, *u*(*ẑ*_*i*_) represents the proportion of MCMC iterations where the estimate of *z*_*i*_ was not the posterior MAP estimate *ẑ*_*i*_. For nodes that share many edges with other nodes within their respective community, i.e., those that are highly typical of their community, the uncertainty measure should be low. Meanwhile, for nodes that share edges with nodes outside of their respective community, the uncertainty measure should be high, as these nodes will likely be assigned to other communities intermittently over the course of the MCMC estimation. In this way, we may augment the cluster labels obtained by the SBM with quantification of our level of confidence in them—a significant advantage over other non-model-based clustering methods.

In addition to uncertainty quantification, we may similarly use the MCMC draws ${\hat{\text{z}}}^{\left(1\right)},...,{\hat{\text{z}}}^{\left(S\right)}$ to conduct *continuous phenotyping*, or the ranking of subjects based on their affinity toward a certain phenotype. For example, in our context of assigning rats to vulnerable and resilient phenotypes using the SBM, we may also provide a continuous measure of affinity toward the vulnerable phenotype for each rat that can be used to rank rats within clusters. In this setting, let cluster *k*_*v*_ ∈ {1, 2, …, *K*} be the cluster annotated as vulnerable for opioid dependence. For each rat *i* = 1, …, *n*, we define the continuous phenotype vulnerability score *v*(*i*) as $\overset{S}{\underset{s=1}{\sum }}I\left({ẑ}_{i}^{\left(s\right)}=k_{v}\right)/S$, i.e., the proportion of MCMC iterations in which rat *i* is assigned to cluster *k*_*v*_.

### Software Implementation

For convenient implementation of the workflow proposed throughout section Materials and Methods, we developed “mlsbm,” an efficient and user-friendly R package for the identification of sub-populations in network data (27). The mlsbm package is freely available for download from the Comprehensive R Archive Network (28) (https://cran.r-project.org/package=mlsbm). The mlsbm package includes robust documentation to facilitate applications to a variety of clustering tasks.

### Comparison to Alternative Approaches

We sought to assess the performance of the SBM clustering workflow relative to alternative clustering approaches, we applied five popular clustering algorithms, namely the Louvain, walktrap, hierarchical clustering, K-means, and DBSCAN algorithms. The Louvain (29) and walktrap (30) algorithms, like the SBM, are network-based methods that operate on the nearest neighbors network described in section Similarity Network Construction. The Louvain algorithm seeks to maximize the modularity of the graph, a measurement of the strength of clustering structure of a graph relative to randomly generated graphs. The walktrap algorithm uses random walks on the nearest neighbors graph to find the most densely connected sub-graphs, i.e., clusters, within the graph. Hierarchical clustering (11) is a “bottom up” approach that iteratively merges the most similar observations into clusters to form a tree structure that can be used to produce cluster labels for a pre-specified value of *K*. K-means (10) and DBSCAN (31) seek to place boundaries around observations in high-dimensional space such that the data points within boundaries, i.e., clusters, are more similar than those across boundaries. While these approaches are commonly used, they lack the inferential benefits of the SBM such as the ability to choose *K* using model fit criteria and provide uncertainty quantification in addition to cluster labels.

## Results

**Figure 1:**
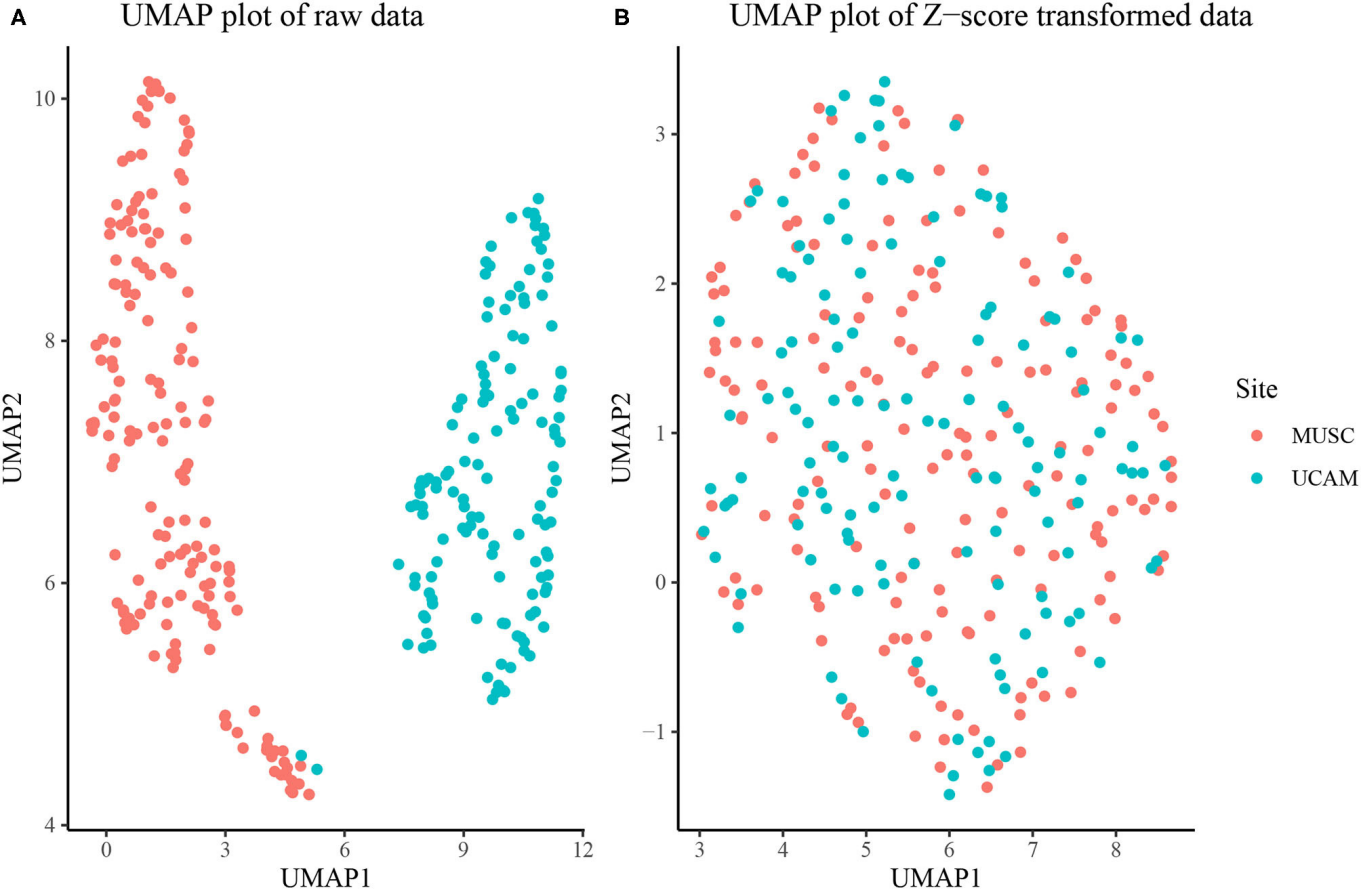
**(A)** UMAP dimension reduction of behavioral measures before site-specific z-scoring shows significant batch effect of study site (MUSC vs. UCAM). **(B)** UMAP dimension reduction after site-specific z-scoring shows adjustment for study site batch effect.

The overall sample was composed of *N*_*m*_ = 238 males and *N*_*f*_ = 213 females. The MUSC study site contributed 243 rats, while the UCAM study site contributed 208. As seen in Figure 1A, the MUSC and UCAM cohorts exhibit clear separation on the 2-dimensional UMAP space, indicating the potential of study site to act as a confounding variable in our analysis, and preventing simultaneous analysis of rats from both cohorts. The site difference is also apparent in Supplementary Figure 6, where in spite of substantially overlapping populations, the MUSC site shows higher mean values than the UCAM site in each of the traits quantified, except for escalation, suggesting a location shift batch effect present between study sites. In Figure 1B, we present the 2-dimension UMAP embedding of the concatenated *z*-score transformed data set, in which no distinguishable separation exists between the MUSC and UCAM rats. Hence, the site-specific z-scoring approach detailed in section Batch Correction for Multi-Site Samples was able to effectively remove the site-specific batch effect from the data.

**Figure 2:**
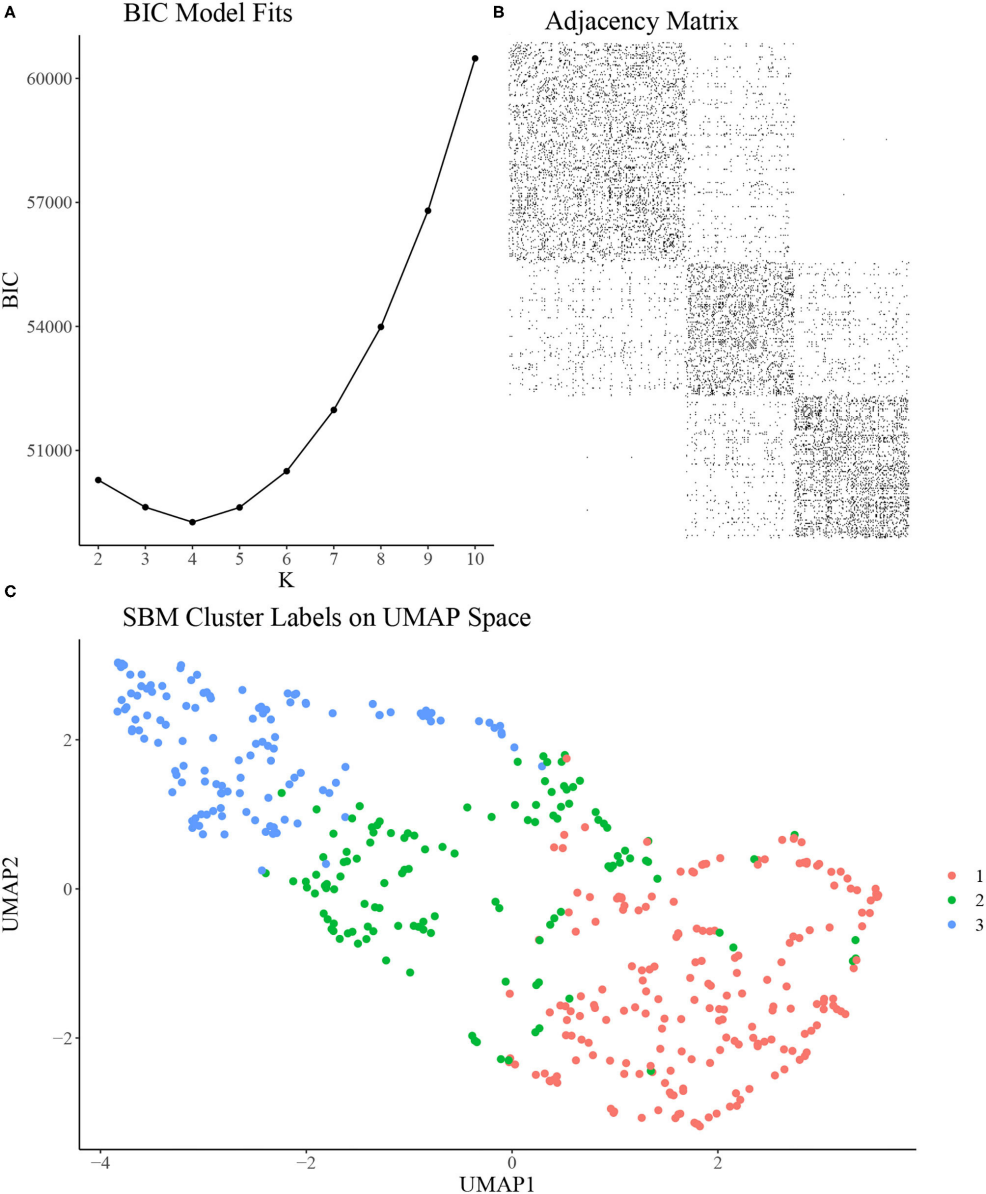
**(A)** Bayesian Information Criterion (BIC) from SBMs fit with a range of *K*. *K* = 3, 4, 5 seem to provide similarly optimal fit in terms of BIC. **(B)** Adjacency matrix of inferred clusters from the SBM using *K* = 3 clusters. **(C)** UMAP reduction of behavioral measurements colored by inferred cluster labels from the SBM using *K* = 3 clusters.

To construct the rat-rat similarity network, we computed the Euclidean distance between each pair of rats using the 7 variables discussed in section Experimental Methods and then formed an adjacency network where each rat was connected to its 21 most similar rats. We applied the SBM clustering analysis described in section Stochastic Block Model to the analysis of *N* = 451 rats. To choose the most appropriate number of clusters *K*, we fit the SBM to the adjacency network for a range of *K* from *K* = 2, …, 10. We ran each model for 10,000 MCMC iterations and discarded the first 1,000 iterations as burn-in, resulting in a total run time of under 4 min for each model using a single 4.7 GHz Intel i7 processor. Using BIC, we found that *K* = 3, 4, 5 provided approximately equal goodness of fit, with *K* = 2 or *K* > 5 provided relatively poor fit (Figure 2A). As such, we chose *K* = 3 to provide the most parsimonious representation of the data and to assess the vulnerable, intermediate, and resilient sub-type hypothesis discussed in section Introduction. An adjacency matrix with rows and columns sorted by inferred cluster indicators from the 3 cluster model is shown in Figure 2B. Figure 2C shows the SBM estimated cluster labels on UMAP space. In Table 1, we present the distribution of two covariates of interest across the three inferred clusters, namely sex and study site. We find a significantly skewed distribution of sex across clusters, with a female bias in cluster 1 and a male bias in cluster 3 (3-sample normal proportion test *p* < 0.0001), while the distribution of study site across inferred clusters is more uniform (3-sample normal proportion test *p* = 0.601).

**Table 1:** Distribution of sex and study site across clusters.

**Cluster**	**% Female (*N*)**	**% UCAM (*N*)**
1: Vulnerable (*N* = 200)	58.5 (117)	44.5 (89)
2: Intermediate (*N* = 122)	47.5 (58)	50.0 (61)
3: Resilient (*N* = 129)	29.5 (38)	45.0 (58)

**Figure 3:**
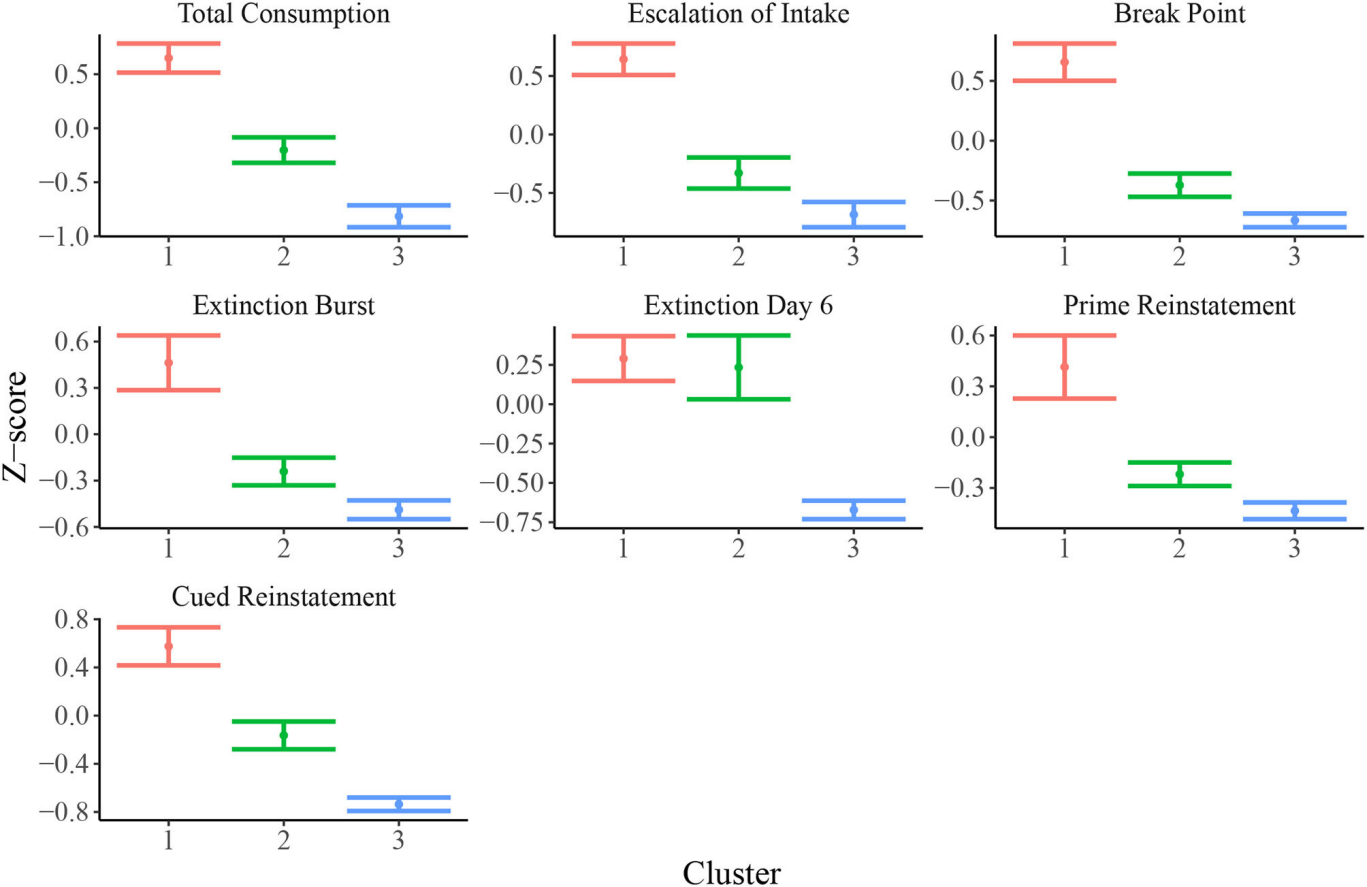
Means and 95% confidence intervals for relevant behavioral measures (z-scored) in each cluster. Distributions of z-scored behavioral variables indicate evidence for vulnerable (cluster 1; *N* = 200), intermediate (cluster 2; *N* = 122), and resilient (cluster 3; *N* = 129) sub-populations.

Figure 3 shows empirical means and 95% *z* confidence intervals for each of the 7 selected behavioral measures across each of the inferred clusters from the SBM. Notably, each cluster appears to show clear separation in most of the behavioral variables. For instance, the total heroin consumption was highest in cluster 1 and lowest in cluster 3, with cluster 2 falling in between clusters 1 and 3, and all 95% confidence intervals not overlapping. Similarly, cluster 1 demonstrated a more rapid escalation of heroin intake relative to clusters 2 and 3. We quantified the difference between clusters by fitting a one-way ANOVA for each of the 7 behavioral measures vs. the SBM cluster indicators. We conducted a global *F*-test for mean differences among groups. F-statistics and associated *p*-values are displayed in Table 2. Distributions of raw behavioral measures in each cluster are shown in Supplementary Figure 1, where the same pattern persists as with standardized variables. We observed qualitatively consistent results in site-specific analyses (Supplementary Figure 4). We quantified this observation through use of the adjusted Rand index (ARI) between each site-specific analysis and the integrated analysis, which revealed high correspondence between each site-specific analysis and the integrated analysis (MUSC ARI = 0.43; UCAM ARI = 0.54).

**Table 2:** ANOVA global F-statistics and associated *p*-values for each behavioral measure.

**Variable**	**F-statistic**	***P*-value**
Total consumption	283.8	<0.0001
Escalation of intake	220.7	<0.0001
Break point	221.6	<0.0001
Extinction burst	94.78	<0.0001
Extinction day 6	77.12	<0.0001
Prime reinstatement	72.36	<0.0001
Cued reinstatement	200.6	<0.0001

**Figure 4:**
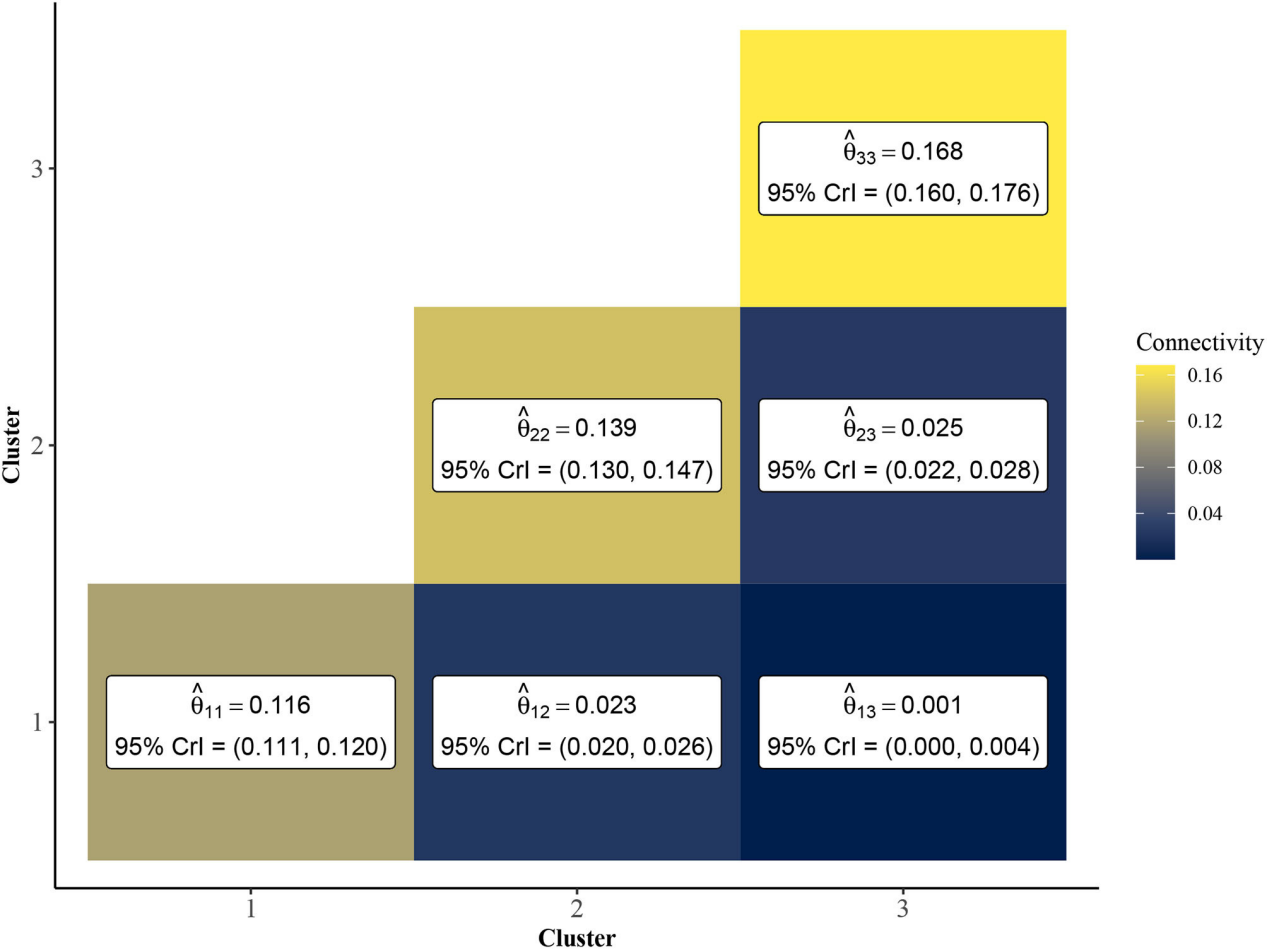
Point estimates and 95% credible intervals of cluster connectivity parameters **Θ**. The SBM estimates higher values for within-cluster connectivity parameters, θ_11_, θ_22_, and θ_33_, which is indicative of an assortative community structure. Thus, rats within the same community are expected to have significantly higher similarity than rats of different clusters. Clusters 1 and 3 are most dissimilar as evidenced by lower values of ${\hat{\theta }}_{13}$ relative to ${\hat{\theta }}_{12}$ and ${\hat{\theta }}_{23}$.

To further investigate the vulnerable, intermediate, and resilient sub-type hypothesis, we leveraged the inferential abilities of the Bayesian SBM to infer the similarity among rats from each cluster. Specifically, by investigating the posterior distribution of the elements of the matrix **Θ**, we may characterize the similarity among rats within and between each of the three clusters. In Figure 4, we show a heatmap of posterior means and 95% Bayesian credible intervals for θ_11_, θ_22_, θ_33_, θ_12_, θ_13_, and θ_23_. We found that the estimated values of the within-cluster connectivity parameters θ_11_, θ_22_, θ_33_ were found to be significantly higher than those of the between-cluster parameters θ_12_, θ_13_, and θ_23_. In fact, cluster 1, which had the weakest estimated within-cluster connectivity (${\hat{\theta }}_{11}=0.116$), was still over four times more densely connected than the highest between-cluster connection, which was shared between clusters 2 and 3 (${\hat{\theta }}_{23}=0.025$). This is indicative of strong assortative community structure in the rat-rat similarity network, in which rats of the same community are more likely to be correlated in terms of behavioral measurements than rats of differing communities. Further, Figure 4 shows that clusters 1 and 3 were the most dissimilar, with cluster 2 serving as an intermediate cluster.

**Figure 5:**
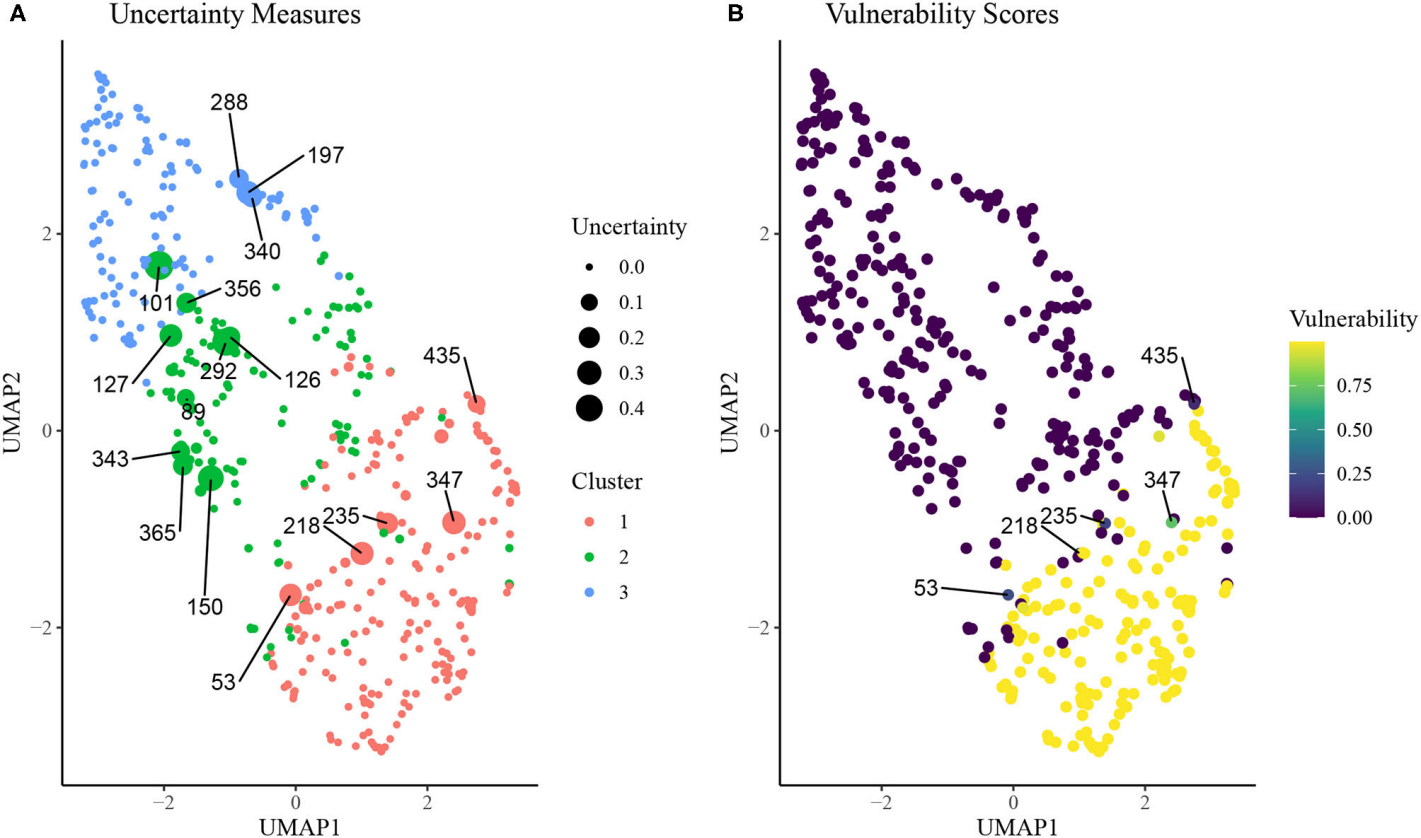
**(A)** Uncertainty scores of cluster assignment for each rat on UMAP space. Animal IDs are given for subjects with uncertainty measure above 0.10, which is indicative of at least 10% of MCMC iterations spent in a cluster other than the final inferred cluster. **(B)** Vulnerability scores for each rat on UMAP space. Animal IDs were shown for subjects with uncertainty above 0.10 and vunerability <0.90.

In Figure 5, we plot results from the uncertainty measure and continuous phenotyping analysis presented in section Continuous Phenotyping. Figure 5A plots the cluster assignments on UMAP space, where each point is sized proportionally to its uncertainty measure of cluster assignment (larger points imply higher uncertainty). We label the ID of each rat that featured an uncertainty measure above 0.10, corresponding to rats that spent at least 10% of the post burn-in MCMC iterations from the *K* = 3 SBM in a cluster other than the cluster it was assigned to by the MAP estimate $\hat{\text{z}}$. A number of interesting patterns emerge from this uncertainty analysis. First, we find that rats with higher uncertainty tend to be located near borders between clusters on the UMAP space. Interestingly, rat 101, which was assigned to cluster 2 but is surrounded in UMAP space by rats in cluster 3, featured high uncertainty. Meanwhile, several cluster 2 rats were surrounded by cluster 1 rats in the UMAP space but featured low uncertainty.

Figure 5B displays results from the continuous phenotyping analysis, wherein cluster 1 was annotated as the vulnerable cluster (Figure 3) and chosen as the phenotype of interest. We computed the vulnerability score of each rat as the proportion of post burn-in MCMC iterations from the SBM that were spent in cluster 1. We labeled the IDs of the most interesting rats: those with uncertainty measures above 0.10 but vulnerability measures less than 0.90. These rats were located on the border between the intermediate cluster 2 and the vulnerable cluster 1, indicating higher propensity toward opioid dependence than other rats in cluster 2. These results demonstrate the ability of continuous phenotyping to augment the clustering results of the SBM to allow for disambiguation of within-cluster differences between subjects.

**Figure 6:**
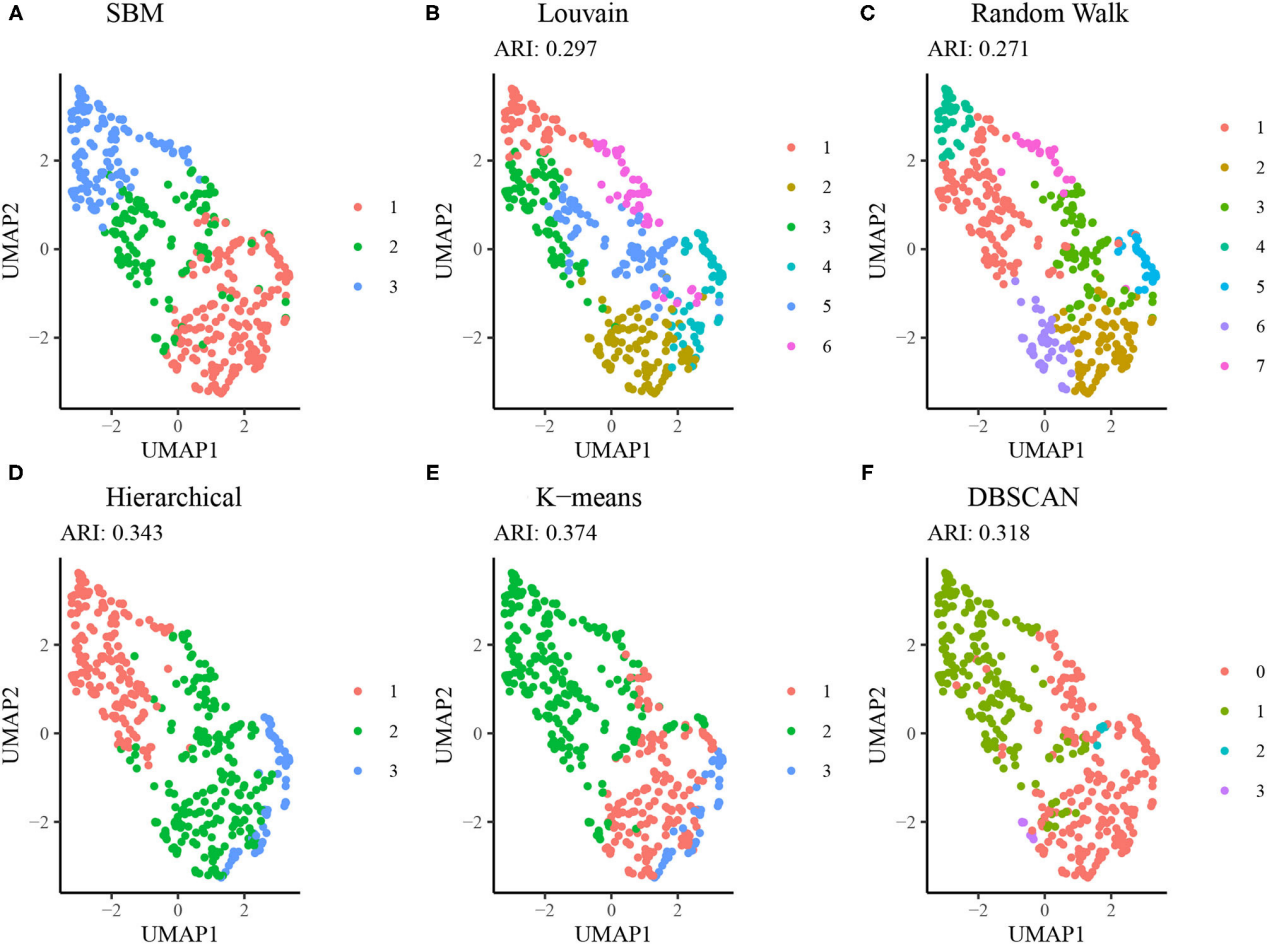
Comparison of SBM performance relative to alternative methods. **(A)** Clustering results from the SBM using *K* = 3. **(B)** Clustering results from the Louvain algorithm (no tuning parameters available). **(C)** Clustering results from the Walktrap algorithm using random walks of length 4. **(D)** Hierarchical clustering results using a dendrogram cut at *K* = 3. **(E)** K-means clustering results using the Hartigan-Wong method and *K* = 3. **(F)** DBSCAN clustering results using a radius of 0.8 and minimum neighborhood size of 5.

Figure 6 displays results from alternative clustering methods as described in section Comparison to Alternative Approaches. The network-based clustering algorithms such as Louvain and walktrap algorithms tended to produce a larger number of clusters, each smaller in size relative to the SBM. Due to this, the agreement between the results from these methods and those from the SBM is low (ARI < 0.30). Both the hierarchical clustering method using squared Ward dissimilarity (32) and the K-means algorithm resulted in moderate agreement with the SBM (ARI = 0.343 and 0.374, respectively), while the DBSCAN algorithm yielded a 4 cluster result using default parameters, two of which were sparsely populated. These results suggest the SBM is best suited to addressing the research question at hand.

In addition to validating the capacity of the SBM to create three sub-populations of rats with high, intermediate and low responding for seven heroin associated behavioral traits, we evaluated how the sub-populations compare in terms of weight, site and cohort differences. Supplementary Figure 7 shows that between sites proportionally equivalent numbers of rats were assigned to each sub-population between the two testing site, and when analyzing between cohorts of rats within each site we found that assignment into sub-populations was equivalent across cohorts at the MUSC site, but that differences existed at the UCAM site. Also, because all the behavioral traits involved the same operant response (active lever pressing), we examined whether any traits within each sub-population were correlated using a Pearson's linear correlation statistic. Supplementary Figure 3 shows the Pearson's coefficient for each trait comparison within each sub-population, which reveals that only Extinction Day 6 and Cued reinstatement were linearly correlated within each cluster. Otherwise, there was no consistent trait correlation across the three sub-populations. The lack of linear relationship between traits within the clusters is also revealed in Supplementary Figure 8, which shows the z-scored behavioral responses for all rats in cluster 1 with a selection of rats highlighted for descriptive purposes. Note that rats need not be high responders in all traits to be identified in the cluster 1 sub-population. These differences between clusters and the overall low levels of linear correlation between traits supports exploring the SBM non-linear clustering approach described here as a means to identify non-linear relationships between multiple traits and thereby identify high (vulnerable) and low (resilient) heroin responding sub-populations. Finally, Supplementary Figure 9 shows that equivalent weight gains occurred before and after completing the behavioral testing between each sub-cluster.

## Discussion

In this paper, we developed a comprehensive framework for the descriptive analysis of behavioral sub-populations, and applied it to the cohort of 451 outbred rats subject to heroin self-administration exposure. We discovered the presence of batch effects between the two study sites that contributed to this cohort, and we corrected for these effects using study-site specific z-scoring. Seven behavioral measures were chosen to characterize the vulnerability of each rat to forming opioid dependence. Taken together, these measures quantified three important aspects of dependence: drug-taking, refraining and seeking behaviors. Using these measures, we then converted the multidimensional behavioral data into a rat-rat similarity network, which allowed for investigation of distinct communities within the overall network.

We chose the Bayesian stochastic block model, a statistical model for network data, for investigation of behavioral sub-populations within this cohort. We used the model fit criterion BIC to choose a subset of best fitting models in terms of number of communities. Of this best fitting subset, we chose the three cluster model as it offered the best balance between optimizing statistical and biological criteria. Using ANOVA global *F*-tests, we found significant separation between clusters in terms of each of the seven behavioral measures. Additionally, investigation of average trends across clusters in each behavioral measure allowed us to annotate vulnerable, resilient, and intermediate sub-groups with high confidence. Using the community connectivity parameters inferred by the SBM, we described the relative similarity between clusters, with the vulnerable and resilient clusters each displaying similarity to the intermediate cluster but very little similarity to one another.

To augment the discrete community labels obtained from the SBM, we developed an uncertainty measure, which uses samples from the posterior distribution of the cluster labels to estimate our confidence in the inferred community structure. We also implemented continuous phenotyping to investigate heterogeneities within clusters in terms of vulnerability to opioid dependence. We found a subset of intermediate vulnerability animals who featured relatively high affinity toward the vulnerably cluster, providing candidate animals for further investigation of the differences between vulnerable and resilient animals. Finally, we developed “mlsbm,” an efficient and robust R package for implementation of our proposed clustering workflow. The mlsbm package is publicly available through CRAN (https://cran.r-project.org/package=mlsbm) for use in future behavioral studies.

The SBM analysis identified three behaviorally distinct populations of rats that varied based on their apparent vulnerability to OUD. OUD is a complex and multi-symptomatic disorder, making it imperative to understand how various behaviors over the course of addiction interact with one another to confer vulnerability vs. resiliency. Results indicate that individuals more vulnerable to OUD exhibit higher lever pressing across the behavioral tasks, but largely not in a linear manner (Supplementary Figure 3). Thus, in the SMB, it is the non-linear interaction between several variables that ultimately results in differences between clusters. This is illustrated in Supplementary Figure 8, showing how all animals in cluster 1 (vulnerable cluster) vary across the seven traits we used for modeling. Highlighted are examples of three rats each showing a distinct high and low z-score profile depending on the traits. For example, not all rats in the vulnerable cluster had high heroin consumption, although the mean consumption for this cluster was greater than for the other two clusters (Figure 3).

Both males and females were used in this study, and we found sex differences in cluster composition with females more represented in Cluster 1, and males in Cluster 3. These data align with what is observed in humans, as females both acquire and maintain higher levels of drug use, and relapse more often, than males across several classes of drugs, including heroin (33). This finding further bolsters the potential translational validity of this model in assessing OUD vulnerability. However, a deeper analysis of translational validity requires future studies where traits determined prior to heroin exposure that predict OUD vulnerability in humans can be evaluated to determine if they predict which cluster a rat will enter. For example, levels of impulsivity, novelty-induced locomotor behavior and attributing incentive salience to a reward-paired cue have all been show to predict relapse propensity [for review see (34)]. Moreover, measuring behaviors of drug seeking after obtaining the heroin measures can be used as covariates to further validate cluster allocation by the SBM model. For example, the model would predict that cluster 1 rats would more compulsively seek heroin in the presence of punishment than cluster 3 subpopulations. Also, identifying these three distinct phenotypes using this model allows for further characterization of individual variation in the neurobiological mechanisms and genetic background underlying OUD vulnerability. Finally, we plan to develop an interactive web application using the SBM model to analyze a variety of network-based data sets without the need for programming experience in R, thereby allowing other laboratories to evaluate a variety of network-based data sets for subpopulations of animals and humans that may be more vulnerable or resilient to developing SUDs or other neuropsychiatric disorders.

## Data Availability Statement

The datasets presented in this study can be found in online repositories. The names of the repository/repositories and accession number(s) can be found below: The R package mlsbm is publicly available from the Comprehensive R Archive Network (https://cran.r-project.org/package=mlsbm). The behavioral data used in this paper are not readily available due to ongoing data collection, which is implemented as part of the ongoing NIH-funded research project. Please contact the corresponding author for any inquiry related to the behavioral data.

## Ethics Statement

All experimental procedures were approved by the Institutional Animal Care and Use Committee at the Medical University of South Carolina and by the Italian Ministry of Health (approval 1D580.18). Procedures abided by the National Institute of Health Guide for the Care and Use of Laboratory Animals and the Assessment and Accreditation of Laboratory Animals Care, as well as the European Community Council Directive for Care and Use of Laboratory Animals.

## Author Contributions

Statistical modeling, software development, and data analyses were conducted by CA and DC. The behavioral experiments were designed by NC, BK, MU, LW, GH, RC, and PK. All behavioral experimental procedures were conducted by BK, NC, VL, AC, and AR. This manuscript was written by CA, BK, NC, RC, PK, and DC. All authors contributed to the article and approved the submitted version.

## Funding

This work was supported in part by NIH/NIDA grant U01-DA045300, NIH/NIGMS grant R01-GM122078, NIH/NCI grant R21-CA209848, and NIH/NIDA grant T32-DA007288.

## Conflict of Interest

The authors declare that the research was conducted in the absence of any commercial or financial relationships that could be construed as a potential conflict of interest.

## Publisher's Note

All claims expressed in this article are solely those of the authors and do not necessarily represent those of their affiliated organizations, or those of the publisher, the editors and the reviewers. Any product that may be evaluated in this article, or claim that may be made by its manufacturer, is not guaranteed or endorsed by the publisher.
