# Supplementary material for: Network-Based Discovery of Opioid Use Vulnerability in Rats Using the Bayesian Stochastic Block Model
Source: Front Psychiatry. 2021 Dec 17;12:745468. doi: 10.3389/fpsyt.2021.745468 (PMC8718996; doi:10.3389/fpsyt.2021.745468)
Supplement: Supplementary file 1 [file Data_Sheet_1.PDF]

Supporting Information for "Network-Based Discovery of Opioid Use Vulnerability in Rats Using the Bayesian Stochastic Block Model" by

Carter Allen [1,†], Brittany N. Kuhn [2,†], Nazzareno Cannella [3,†], Ayteria D. Crow [2], Analyse T. Roberts [2], Veronica Lunerti [3], Massimo Ubaldi [3], Gary Hardiman [4], Leah C. Solberg Woods [5], Roberto Ciccocioppo [3], Peter W. Kalivas [2], and Dongjun Chung [1,*]

[1]Department of Biomedical Informatics, The Ohio State University, Columbus, OH, United States.
[2]Department of Neuroscience, Medical University of South Carolina, Charleston, SC, United States.
[3]School of Pharmacy, University of Camerino, Camerino, Italy.
[4]School of Biological Sciences, Queen's University Belfast, Belfast, Northern Ireland, United Kingdom.
[5]Department of Internal Medicine, Wake Forest University School of Medicine, Winston-Salem, North Carolina, United States.
[†] Authors contributed equally to this work.
[*]email: chung.911@osu.edu

Gibbs Sampler

The SBM proposed in the main manuscript allows for closed-form full conditional distributions of all model parameters. Thus, we implemented parameter estimation using the following Gibbs sampling algorithm.

1. Update $\boldsymbol{\pi}$ from its full conditional $(\boldsymbol{\pi}|\mathbf{A}, \mathbf{z}, \boldsymbol{\Theta}) \sim \text{Dirichlet}(a_1, ..., a_n)$, where $a_k = \alpha_k + n_k$, and n_k is the current number of nodes in community k.

2. For $r \leq s = 1, ..., K$, update θ_{rs} from $(\theta_{rs}|\mathbf{A}, \mathbf{z}, \boldsymbol{\pi}) \sim \text{Beta}(1 + A[rs], 1 + n_{rs} - A[rs])$, where $A[rs]$ are the number of observed edges between communities r and s, and $n_{rs} = n_r n_s - n_r I(r = s)$ are the number of possible edges between communities r and s, and $I(r = s)$ is the indicator function equal to 1 if $r = s$ and 0 otherwise.

3. For $i = 1, ..., n$, update z_i from $(z_i|z_{-i}, \mathbf{A}, \boldsymbol{\pi}, \boldsymbol{\Theta}) \sim \text{Multinomial}(\boldsymbol{\rho}_i)$, where $\boldsymbol{\rho}_i = (\rho_{i1}, ..., \rho_{iK})$ and $\rho_{ik} = \pi_k \left(\prod_{j \neq i} \theta_{z_i, z_j}^{A_{ij}} (1 - \theta_{z_i, z_j})^{1 - A_{ij}}\right) \left(\prod_{h \neq i} \theta_{z_h, z_i}^{A_{hi}} (1 - \theta_{z_h, z_i})^{1 - A_{hi}}\right)$.

Supplemental Figures

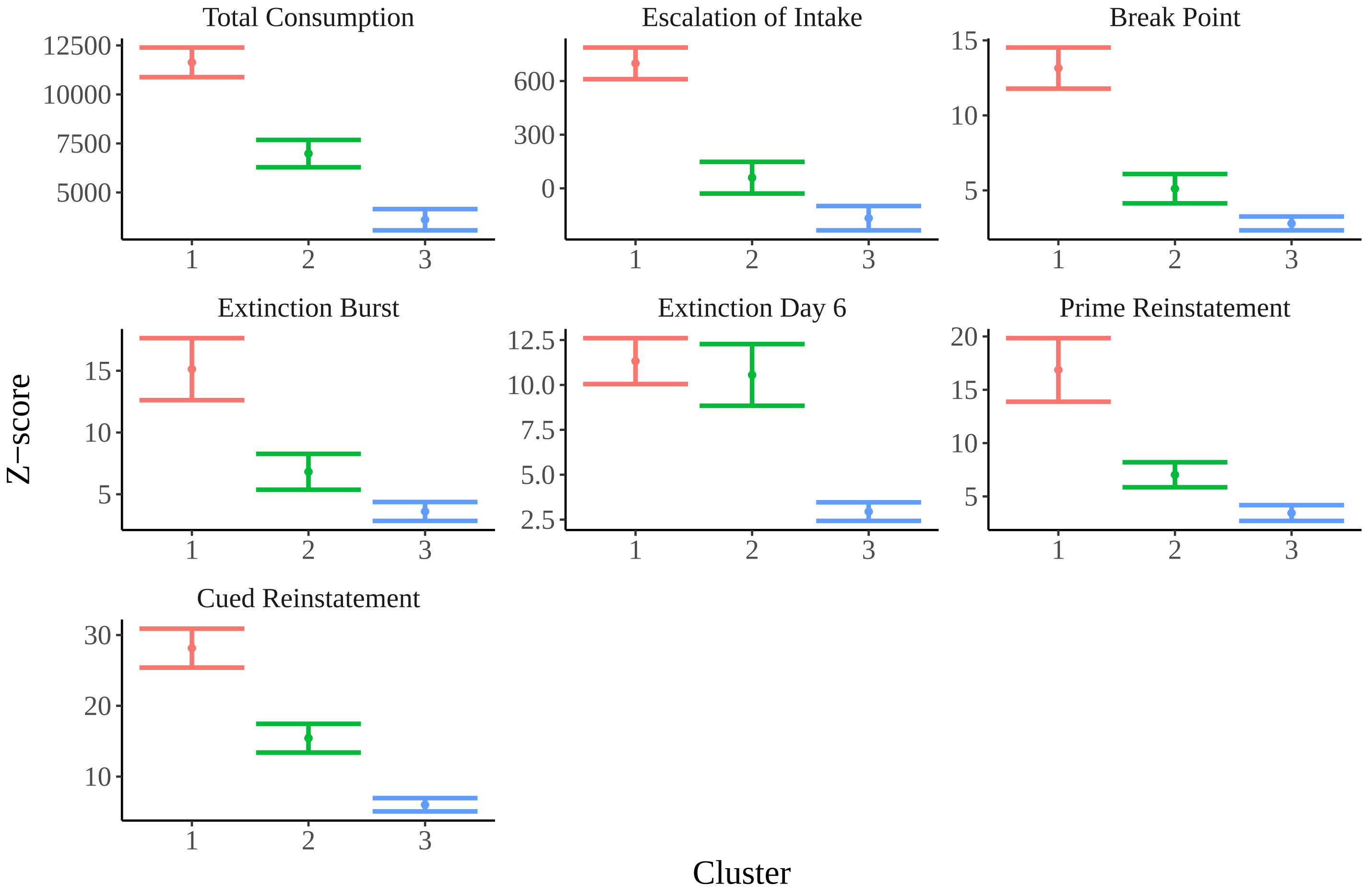

Supplemental Figure 1: Means and 95% confidence intervals for relevant behavioral measures (raw) in each cluster. Distributions of raw behavioral variables indicate evidence for vulnerable (cluster 1; $N = 200$), intermediate (cluster 2; $N = 122$), and resilient (cluster 3; $N = 129$) sub-populations.

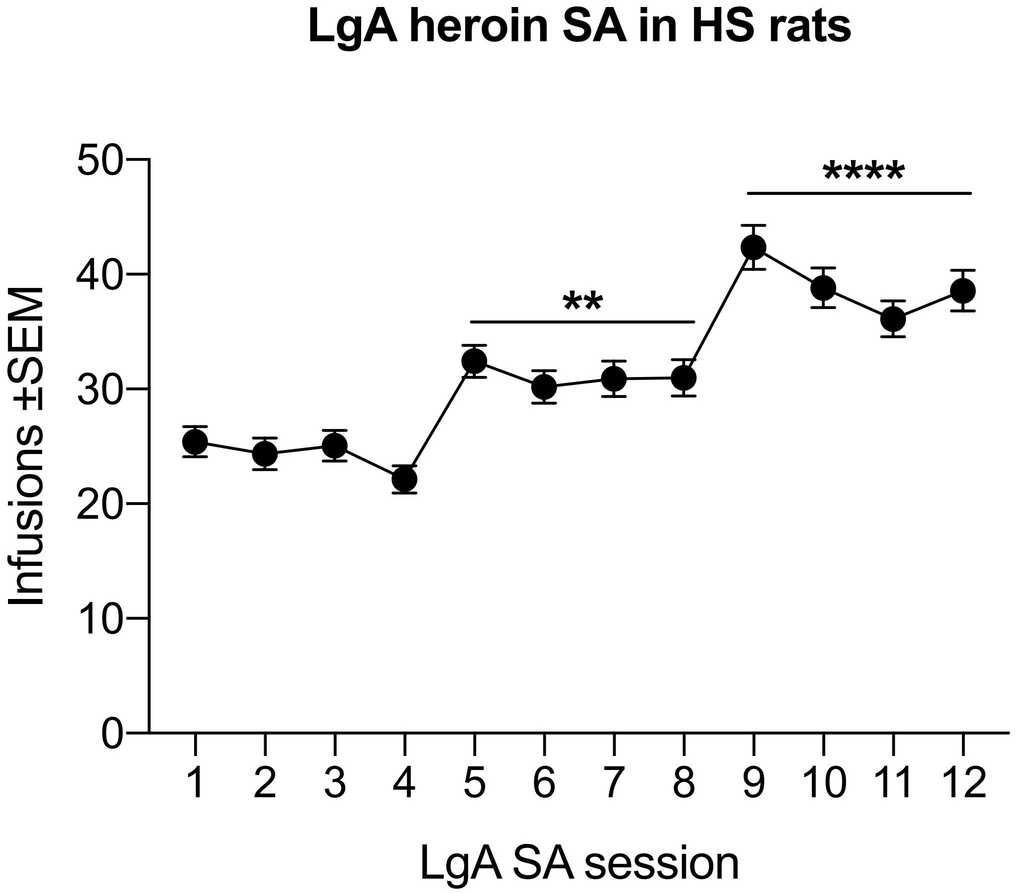

Supplemental Figure 2: Heroin self-administration training under 12h LgA condition in 451 HS rats. Rats acquired self-administration already on the first training session. ANOVA of infusion earned/session found an overall effect of sessions [F(11,4950) = 35.41; p<0.0001]. Dunnet's post hoc analysis was used to compare infusion earned in session 1 with every other session and it revealed that the number of infusions/session remained stable until session 4 and then it escalated starting from session 5. Data are presented as Mean ± SEM. Statistical significance: **p<0.01 and ****p<0.0001 vs LgA SA session 1.

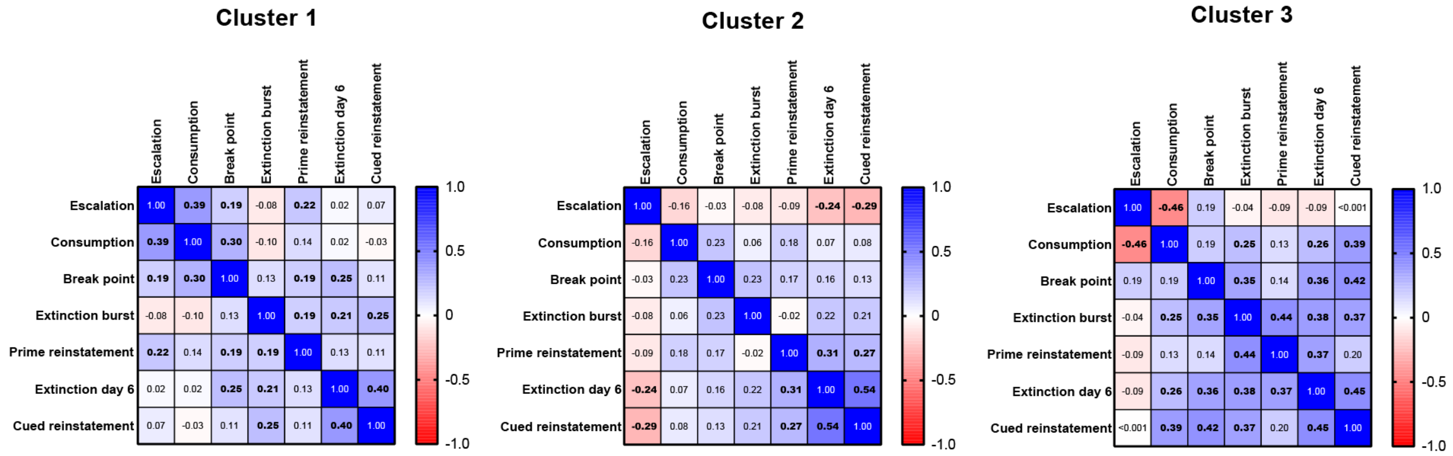

Supplemental Figure 3: Correlations of behaviors within each cluster. Several behaviors were selected for analysis to capture the different phases of opioid use disorder: heroin taking (reinforced behavior), refraining (non-reinforced behavior) and seeking (both reinforced and non-reinforced). Though significant correlations are preset within each cluster, the percent of variance explained by these linear interactions is low. Value within correlation plot are the Pearson r coefficients, and significant interactions ($p<0.05$) are in bold. (Cluster 1, n= 200; Cluster 2, n= 122; Cluster 3, n= 129)

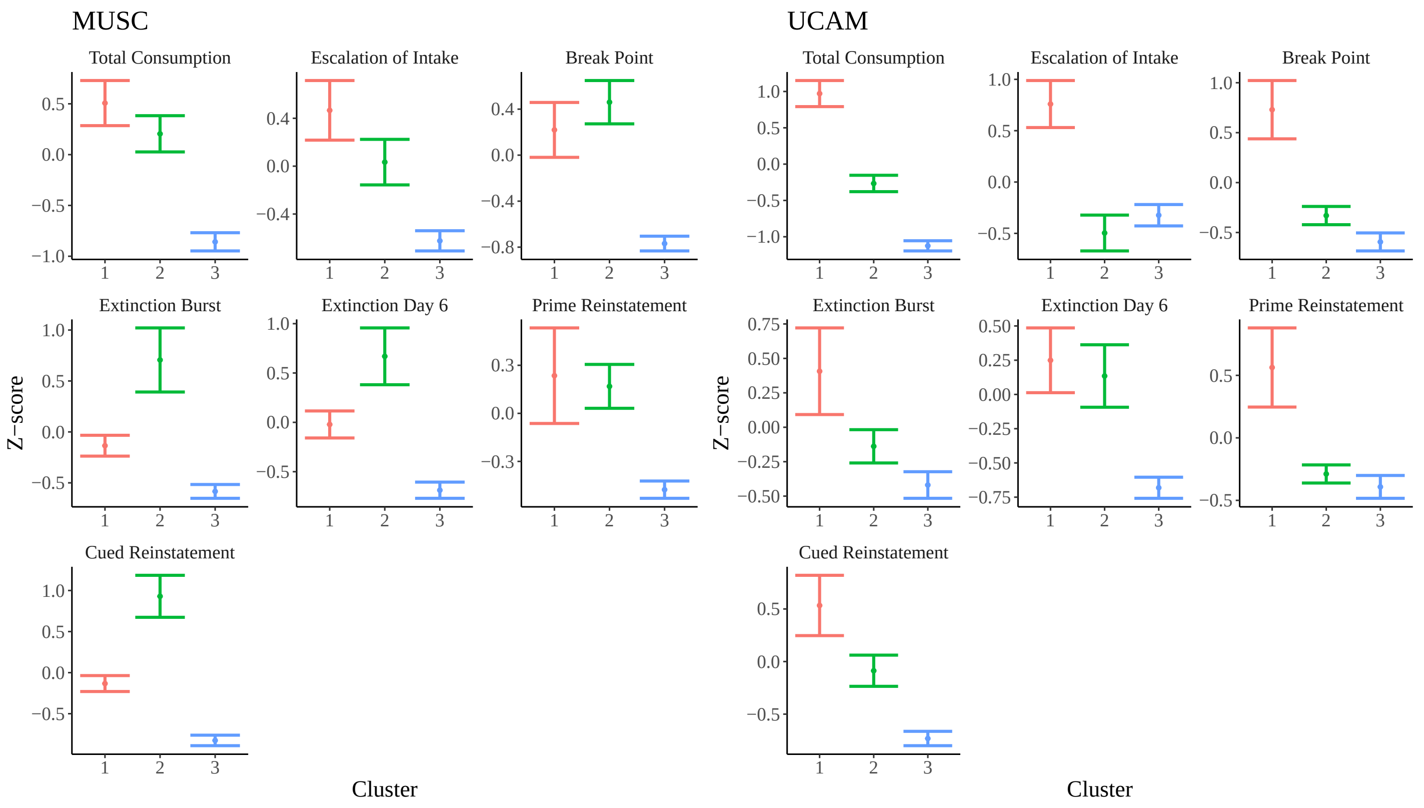

Supplemental Figure 4: Behavioral measures by cluster, where clustering analyses were performed for data from each study site individually.

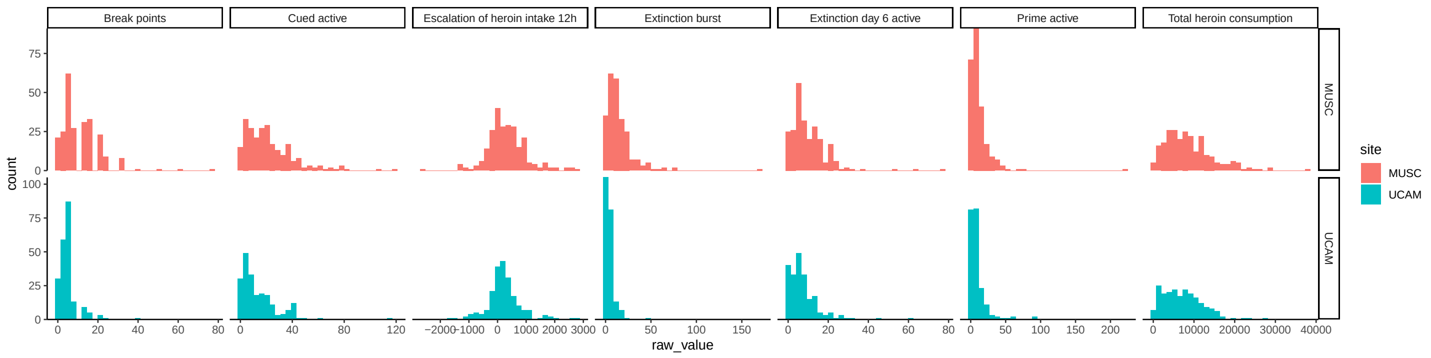

Supplemental Figure 5: Distributions of raw behavioral measures by study site before z-scoring.

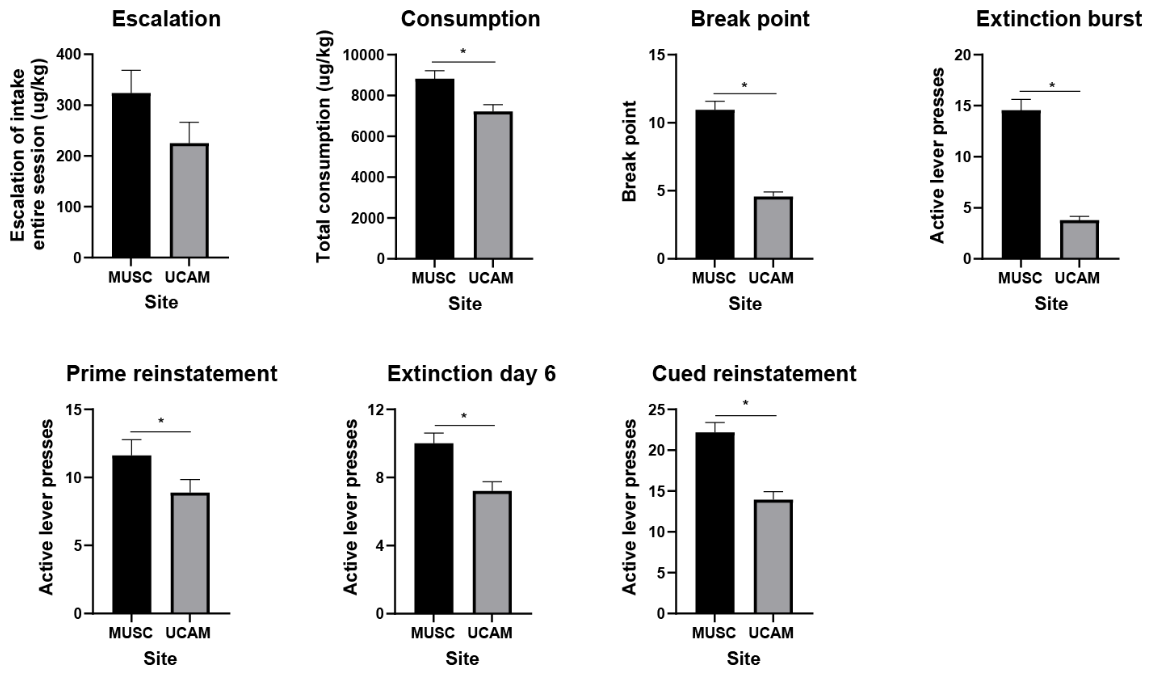

Supplemental Figure 6: Mean ± S.E. comparison between study sites for individual behaviors examined with the network clustering workflow. Note that in all but the escalation trait values were higher from the MUSC site compared with the UCAM site. *$p < 0.05$, using a Student's t-test

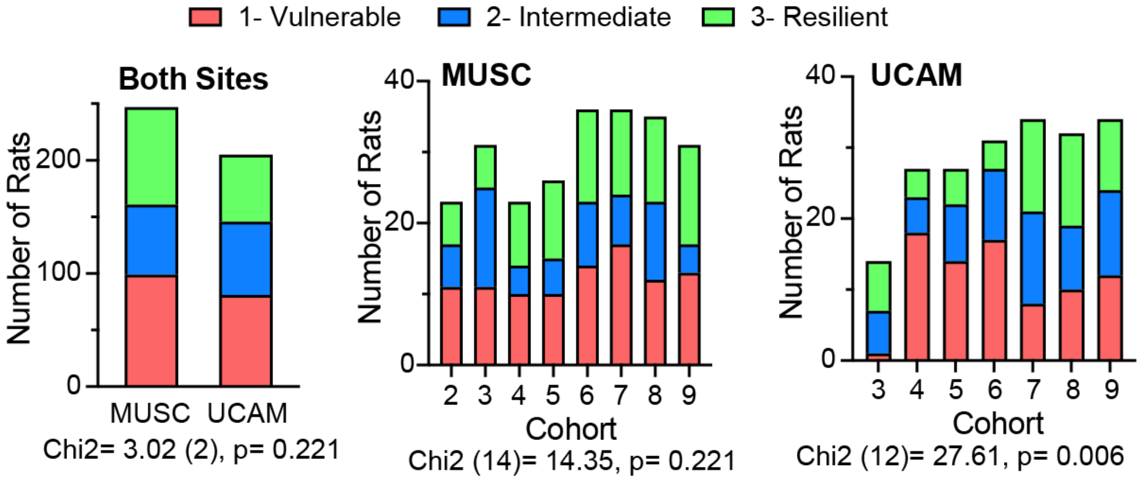

Supplemental Figure 7: Comparisons between cohorts of rat distribution into sub-populations between testing sites. The distributions between sites were equivalent. However, when the sites were examined individually for cohort differences in distribution, the cohorts from the UCAM site did not always distribute equivalently into the three sub-populations.

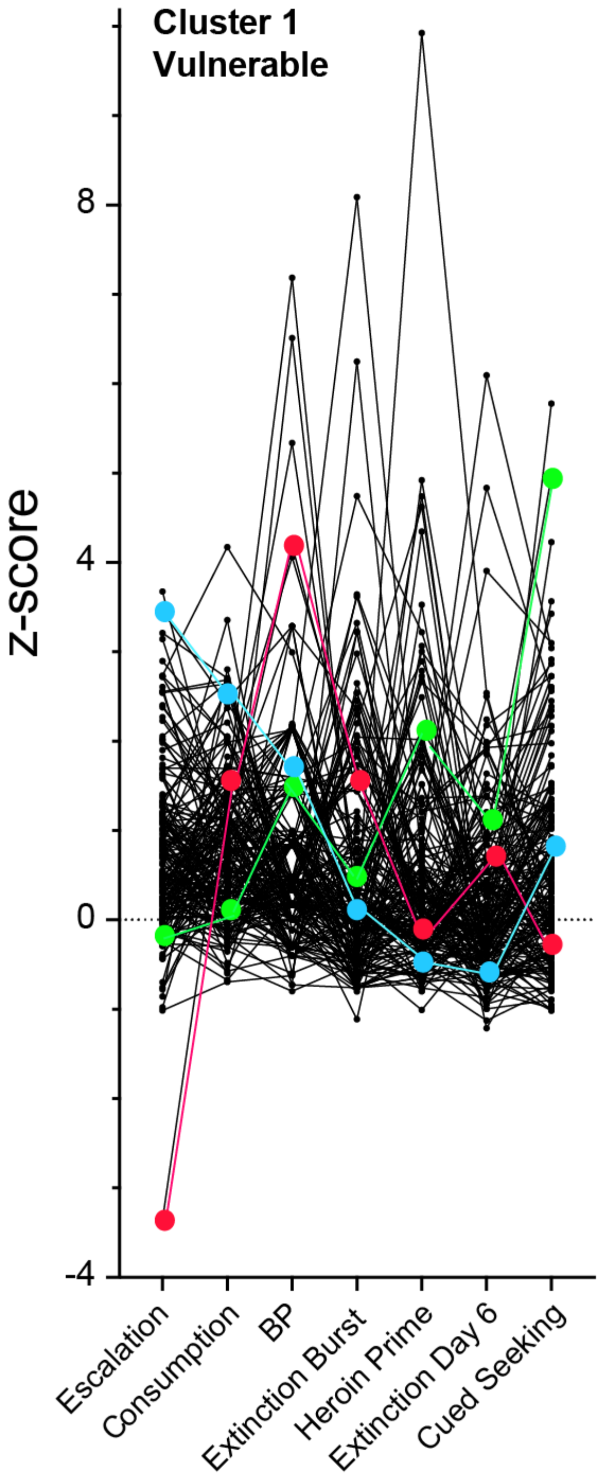

Supplemental Figure 8: Responses of each rat identified to cluster 1 (vulnerable) across each behavior to highlight how rats can demonstrate a high value for different combinations of behavior and be identified into a cluster due to the nonlinear character of the SBM. Three rats are illustratively highlighted: Red- high break point, low escalation; Green- high cued seeking; Blue- high escalation and consumption.

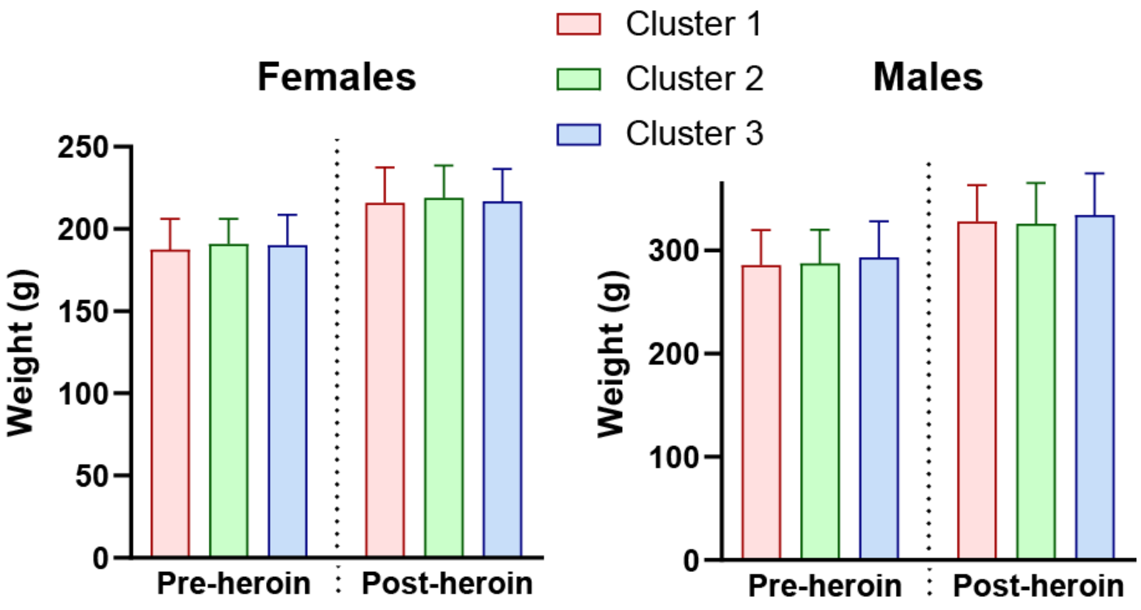

Supplemental Figure 9: Consistent weight gain across cluster before and after rats completed the heroin self-administration protocol. Both males and females showed weight gains between the pre- and post-heroin protocol measurements using a separate two-way ANOVA for each sex, Female- time $F(1,186) = 850.6$, $p<0.001$; Male- time $F(1,204) = 897.4$, $p<0.001$.
